# Spontaneous cSCC Murine Model Shows Limited Response to PD-1 Blockade and Radiation Combination Therapy

**DOI:** 10.3390/cancers18010146

**Published:** 2025-12-31

**Authors:** Tara M. Hosseini, Laura Ho, Tammy B. Pham, Alfredo Molinolo, Riley Jones, David Vera, Andrew Sharabi, Soo J. Park, Theresa Guo

**Affiliations:** 1 Moores Cancer Center, University of California San Diego, La Jolla, CA 92093, USA; 2 Department of Otolaryngology-Head & Neck Surgery, University of California San Diego, La Jolla, CA 92093, USA; 3 Department of Pathology, Moores Cancer Center, University of California San Diego, La Jolla, CA 92093, USA; 4 Department of Radiation Medicine and Applied Sciences, University of California San Diego, La Jolla, CA 92093, USA; 5 Department of Radiology, University of California San Diego, La Jolla, CA 92093, USA; 6 Department of Hematology-Oncology, University of California San Diego, La Jolla, CA 92093, USA

**Keywords:** cutaneous squamous cell carcinoma, immunotherapy, anti-PD1, cSCC, radiation

## Abstract

**Background/Objectives**: Non-melanoma skin cancer, which includes cutaneous squamous cell carcinoma (cSCC), ranks as the 5th most common cancer globally with high morbidity and more total deaths than melanoma despite having a lower mortality rate. While most cSCC cases can be treated with surgery, locally advanced, metastatic, and high-risk cSCC tumors are associated with a worse prognosis with higher rates of recurrence and require multimodality therapy. However, there is limited data on animal models of cutaneous squamous cell carcinoma for the use of combinatory immunotherapy and radiation. **Methods**: In this study, spontaneously generated tumors using DMBA/TPA were treated over three weeks with either IgG control, anti-PD1 antibody monotherapy, 8 Gy of localized radiation, or a combination of anti-PD1 and 8 Gy of radiation followed by anti-PD1 therapy. **Results**: We found that while anti-PD1 therapy showed a trend toward slowed tumor growth compared to controls, this difference was not statistically significant (*p* = 0.0775), with most mice showing continued tumor progression. Preliminary histological analysis suggested that anti-PD1 treatment increased CD8+ T cell infiltration, and the addition of radiation further enhanced CD8+ responses but added greater variability. A pathologic review revealed that irradiated tumors were associated with fibroblastic spindle-like cell morphology. **Conclusions**: This animal model represents a potential preclinical model for studying CSCC with limited responses to immunotherapy to understand potential mechanisms of resistance.

## Simple Summary

The prevalence of cutaneous squamous cell carcinoma (cSCC) globally has increased significantly in the last 40 years. Specifically, locally advanced, metastatic, and high-risk cSCC tumors are associated with a worse prognosis and require multimodality therapy. This study investigated the effectiveness of treatment on spontaneously generated cSCC with both anti-PD1 monotherapy and the combinatorial anti-PD1 immunotherapy with radiation. Anti-PD1 therapy showed a trend toward a slowed progression of cSCC and increased CD8+ expression in preliminary single-specimen analysis.

## 1. Introduction

Cutaneous squamous cell carcinoma (cSCC) is the second most prevalent non-melanoma skin cancer, responsible for approximately 20% of all skin cancers and affecting over one million individuals annually in the United States [1,2]. Recent global studies have highlighted that non-melanoma skin cancer is now responsible for more total deaths than melanoma, despite melanoma’s higher mortality rate [3]. The pathogenesis of cSCC is strongly associated with ultraviolet (UV) radiation exposure, which induces mutagenesis and genomic instability [4,5,6]. Body regions with the highest UV exposure, such as the head, neck, and back, are particularly susceptible to developing cSCC [1]. UV-induced DNA damage contributes to the high rate of genetic mutations characteristic of cSCC, namely mutations in the tumor suppressor gene *TP53* that account for more than half of cSCC cases [4,7]. Other environmental and genetic risk factors include fair skin, older age, male sex, and prior cancer diagnoses [1,5].

The current standard of treatment for localized cSCC is surgical excision, with adjuvant radiation therapy if indicated for high-risk pathologic features [7,8]. However, in high-risk and advanced disease, incomplete excision and aggressive biology can result in recurrent disease with significant morbidity and mortality [7,8,9]. cSCC recurrence occurs in up to 30–40% of patients with high-risk locally advanced disease despite employing multimodal therapies, such as surgery followed by adjuvant radiation or chemotherapy [7]. These drawbacks further underscore the need for novel therapeutic approaches to improve oncologic outcomes.

Over the past decade, immunotherapy has emerged to revolutionize the treatment landscape for non-melanoma skin cancers, including cSCC, basal cell carcinoma, and Merkel cell carcinoma [10,11]. One of the most well-studied checkpoints is the programmed cell death protein 1 (PD-1) pathway, which involves an inhibitory receptor expressed on activated T cells [11,12,13]. T cell-mediated immune regulation plays a critical role in cSCC development, as demonstrated by studies showing that the modulation of UVB-induced regulatory T cells can prevent cSCC establishment [14]. However, once tumors are established, the mechanisms of immunotherapy resistance remain incompletely understood. Recent evidence has demonstrated the high efficacy of anti-PD1 inhibition in the treatment of cSCC, with responses in ~45% of patients with recurrent and metastatic disease [15,16]. Furthermore, in the neoadjuvant setting, up to 50% of patients had a complete pathologic response to the PD-1 immune checkpoint inhibitor cemiplimab, showing its potential for high efficacy [16]. Lastly, recent data has also demonstrated the efficacy of these therapies in the adjuvant setting after surgery and radiation [17].

Despite this promising data, there remain patients that do not respond to immunotherapies. Additionally, there is a lack of biomarkers to clearly identify which patients will or will not respond well to anti-PD1 inhibitors. Therefore, preclinical models that provide a greater understanding of the underlying biology of immune checkpoint inhibitors, particularly for high-risk cutaneous SCC that do not respond, are needed. Herein, we describe the efficacy of anti-PD1 immunotherapy both as a monotherapy and as an adjunct to primary radiation in a murine model of cSCC, as well as the impact of combining radiation with anti-PD1 immunotherapy in this tumor model.

## 2. Materials and Methods

### 2.1. Chemicals and Reagents

For induction of cSCC tumors, we utilized the application of 7,12-dimethylbenzo[a]anthracene (DMBA; Thermo Fischer Scientific Chemicals, Waltham, MA, USA; Cat. #AC408181000) and 12-O-tetradecanoylphorbol-13-acetate (TPA; Thermo Fischer Scientific Chemicals, Waltham, MA, USA; Cat. #10008014), as previously published [18,19]. DMBA and TPA were dissolved in acetone and ethanol, respectively, for tumorigenesis preparation on mice skin. The CD279 variants of anti-mouse PD-1 (Bio X Cell, Lebanon, NH, USA; Cat. #BP0146) and rat IgG2a isotope control (Bio X Cell, Lebanon, NH, USA; Cat. #BP0089) injections were used and diluted in phosphate-buffered saline (PBS) to a dose of 10 mg/kg per individual mouse.

### 2.2. Animals

Six-week-old male and female FVB/NJ mice used in this experiment were obtained from Charles Rivers Lab (Wilmington, MA, USA). All care and usage of animals were approved by the Institutional Animal Care and Use Committee at the University of California, San Diego.

### 2.3. In Vivo Skin Tumorigenesis

The mice (5–6 weeks of age) were shaved on their backs and treated topically on the exposed dorsal skin with 100 μL DMBA. Post-DMBA administration, mice were placed in quarantine cages for 7–9 days. Following the quarantine period, 100 μL of TPA was applied to the same exposed skin twice a week until tumorigenesis.

Tumors were identified based on the following visual morphology: red, raised, thickened skin, and scaly, usually accompanied by a central depression and scabbing. Once treatment was initiated, TPA application was stopped. Complete tumorigenesis in this mouse pool occurred after a range of 2 to 7 months after initial TPA application. All carcinomas and the presence of nodal metastasis were confirmed upon histopathology by a pathologist (AM).

### 2.4. Tumor Treatment via Intraperitoneal (IP) Injections

When mice developed carcinoma that exceeded 6 mm in primary length, they were randomly assigned into one of four designated treatment groups: those receiving an anti-PD-1 treatment or IgG control with or without stereotactic radiation, as described below. Treatment was initiated when tumors reached greater than 9.9 mm in diameter.

Both groups of anti-PD-1 and IgG treatments were administered as an intraperitoneal (IP) injection using an insulin needle according to mouse weight (10 mg/kg). Intraperitoneal injections for mice with viable carcinomas were administered three times a week. Mice received three weeks of intraperitoneal injections for a total of nine injections until sacrifice.

### 2.5. Tumor Measurement and Calculations

Tumor dimensions were measured three times a week at consistent intervals using calipers for the 3-week duration of treatment. Since the cutaneous tumors exhibited lateral spread rather than spherical growth, tumor size was calculated as surface area (height × width, mm^2^) to accurately reflect tumor burden.

Based on our institutional animal care and use committee (IACUC) protocol, mice were sacrificed before that time if tumors reached over 20 mm in diameter or if mice demonstrated severe enlargement of lymph nodes.

For analysis, tumor sizes were normalized to their individual baseline (measurement taken when tumors reached greater than 9.9 mm prior to first treatment injection). Percentage change from baseline is calculated as ((value − baseline)/baseline) × 100.

### 2.6. Histological Analysis—Morphology

The animals were euthanized, and the tumor and lymph nodes were dissected, fixed in Z-Fix, and embedded in paraffin. Five-micron sections were obtained and stained with H&E for histopathological diagnosis.

Sections from tumors with a fibrosarcoma-like spindle-shaped morphology were immunostained with anti-cytokeratin (Anti-wide spectrum Cytokeratin antibody; Abcam, Cambridge, UK; Cat.#ab9377), anti-e-cadherin (E-Cadherin (24E10) Rabbit mAb; Cell Signaling Technology, Danvers, MA, USA; 3195), and anti-vimentin antibodies (Vimentin Rabbit mAb; Abclonal, Woburn, MA, USA; Cat.#A19607) using the ImmPRESS^®^ HRP Anti-Rabbit IgG PLUS Polymer Kit with Peroxidase (Vector Laboratories, Newark, CA, USA; Cat.#MP-7801-15). Tumors exhibiting classical squamous cell carcinoma features and containing non-transformed squamous epithelium were used as positive controls.

### 2.7. Histological Analysis—Multiplex

Two days after treatment completion, mice were sacrificed. Carcinoma samples from both IgG and anti-PD1 treatment groups were harvested and fixed in Zinc Formalin Fixative (Z-fix; Sigma Aldrich, St. Louis, MO, USA; Cat.#Z2902) for 24 h before being transferred into ethanol to fix for another 24 h. After fixation, samples were given to the Biorepository and Tissue Technology Core at UC San Diego, where they were embedded, sectioned, and stained for Multiplex Immunofluorescence visualization.

Multiplex immunohistochemistry was performed on tumors from individual mice (*n* = 1 per treatment group) for DAPI, CD4, CD68, CD8, PAN-CK, PDL1, and CD4 markers by UC San Diego Biorepository and Tissue Technology Core. Histological regions were mapped out by a pathologist (AM) and multiple representative sections of both tumor-involved regions and regions in the periphery of the tumors were identified in each treatment condition for analysis. Fields of view (FOV) encompassing five representative areas (300 µm × 300 µm) of “tumor” and five representative areas of “periphery” within each sample were captured and used to quantify the immune response. DAPI was used to normalize cell counts between groups. Analysis was performed using QuPath software (version 0.5.1).

Due to limited biological replication, these analyses are exploratory and preliminary in nature.

### 2.8. Statistical Analysis

Tumor Size Analysis:

For comparison of tumor sizes (mm^2^) on day 21, statistical analyses were performed on surviving mice at this timepoint. Initial group sizes were PD1 (*n* = 10), IgG (*n* = 8), PD1 + RT (*n* = 4), and IgG + RT (*n* = 3). By day 21, group sizes were PD1 (*n* = 7), IgG (*n* = 6), PD1 + RT (*n* = 2), and IgG + RT (*n* = 3) due to mortality and humane endpoints.

Data was assessed for normality using the Shapiro–Wilk test. Three planned comparisons were performed: (1) PD1 vs. IgG, (2) IgG vs. IgG + RT, and (3) PD1 vs. PD1 + RT. For the PD1 vs. IgG comparison, an unpaired two-tailed *t*-test with Welch’s correction was performed. For the IgG vs. IgG + RT comparison with small sample size (*n* = 3), a non-parametric Mann–Whitney test was performed. The PD1 vs. PD1 + RT comparison could not be performed due to insufficient sample size (*n* = 2) in the PD1 + RT group. Bonferroni correction was applied to adjust for multiple comparisons (adjusted α = 0.017). Statistical significance was defined as *p* < 0.017.

Percentage Change Analysis:

Tumor growth kinetics were analyzed using percentage change from baseline (day 0) for each individual mouse. For each mouse, percentage change was calculated as 100 × (tumor size at timepoint − baseline tumor size)/baseline tumor size. Group means and standard errors of the mean (SEM) were calculated from the individual percentage changes at each timepoint.

For longitudinal comparisons across all timepoints, data was assessed for normality using the Shapiro–Wilk test. Pairwise comparisons at each timepoint (PD1 vs. IGG, PD1 vs. PD1 + 8GY, and IGG vs. IGG + 8GY) were performed using Welch’s *t*-test (for normally distributed data) or Mann–Whitney U test (for non-normally distributed data). Bonferroni correction was applied to account for multiple comparisons across timepoints, and statistical significance was defined as adjusted *p* < 0.05.

For day 21 endpoint comparisons, the same pairwise comparisons were performed using Welch’s *t*-test. No correction for multiple comparisons was applied, as day 21 was a pre-specified single endpoint. Statistical significance was defined as *p* < 0.05.

Histology Analysis:

Normality was assessed using the Shapiro–Wilk test. Pairwise comparisons between individual mice were performed using Welch’s *t*-test, which does not assume equal variances, on FOV-level measurements. *p*-values are reported without adjustment for multiple comparisons, as these represent preliminary observations from single specimens.

Statistical analyses were performed using GraphPad Prism (version 10.6.1), Microsoft Excel, and R (version 4.5.1).

### 2.9. Lymphatic Mapping

Sentinel draining lymph nodes were identified with either lymphazurin or fluorescent-labeled tilmanocept at the time of tumor harvest. For lymphazurin sentinel node identification, 20 µL of lymphazurin was injected dermally into four points of the tumor perimeter. Following this, the lymphatic channel was identified and then used to excise the draining lymph node. Fluorescently labeled tilmanocept was also used in some cases; similarly, a concentration of 0.6 nmol/10 µL was used. Under anesthesia, a total of 40 µL of tilmanocept was injected dermally into four points on the perimeter of the tumor lesion. The mouse was then left awake for 30 min before euthanizing and imaging under Fluobeam. Sentinel nodes were evaluated for presence of metastatic disease confirmed on pathology.

### 2.10. Radiation

For mice selected for radiation treatment, all mice were irradiated with the Precision X-Ray SmART system (Precision X-Ray; North Branford, CT, USA). Mice were individually imaged with cone beam CT scans (CBCT) and the resulting DICOM image files were used for treatment planning. The largest skin cancer lesion was selected as the target lesion for irradiation and set as the isocenter and the treatment plan, including the beam angles and collimator size, and was adapted to a PTV of D90. Lesions were treated with 8 or 12 Gy in a single fraction, depending on treatment group, using 225 kV in 1 fraction. Radiation treatment was given prior to initiation of anti-PD-1 or IgG treatment for selected mice.

## 3. Results

### 3.1. PD1 Treatment Reduces Growth Rate of cSCC Lesions

**Figure 1:**
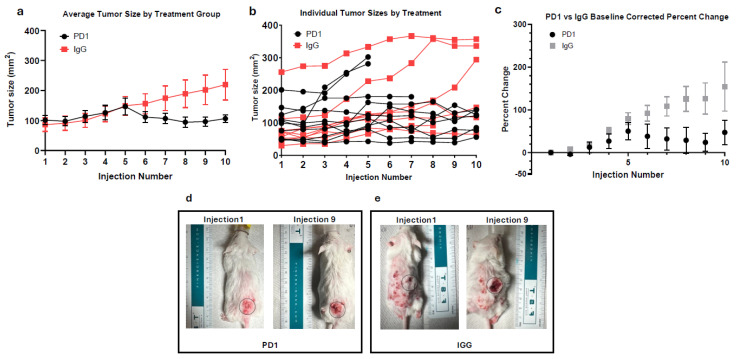
(**a**) The average size of anti-PD1 (*n* = 10) and IgG (*n* = 8)-treated tumors over treatment duration. (**b**) Individual measurements of tumors treated with anti-PD1 or IgG over treatment duration. (**c**) Average baseline-corrected percentage change of anti-PD1 and IgG-treated tumors. Error bars represent SEM. (**d**) Visual progression of representative cSCC lesion after 3 weeks of anti-PD1 treatment. Grey circle represents area of interest with standardize diameter of 17 mm. (**e**) Visual progression of representative cSCC lesion after 3 weeks of IgG treatment. Grey circle represents area of interest with standardize diameter of 17 mm.

In Figure 1a, anti-PD1 and IgG-treated tumors showed similar growth until day 9 (fifth injection), after which anti-PD1-treated tumors showed a slight decrease in tumor size before stabilizing. By the end of treatment, the anti-PD1 tumors increased ~1.1-fold from baseline, while the IgG-treated tumors steadily grew ~2.6-fold. Although tumors in the anti-PD1 group showed slower growth, the change from baseline was not statistically significant (*p* = 0.0775), likely due to the large variation of responses to treatment, small sample sizes, and termination of treatment due to ethical endpoints (tumor size > 20 mm, severe ulceration, or disease-related mortality). Therefore, this data suggests a trend toward reduced tumor growth with anti-PD1 treatment, but definitive conclusions about therapeutic benefit require further validation.

As shown in Figure 1b, none of the treated mice achieved substantial tumor regression. To quantify treatment response, we compared each mouse’s maximum tumor size to its final tumor measurement. In the anti-PD1 group, 40% of mice had ≥10% reduction from their peak tumor size, while the remaining 60% were the largest at their final measurement, and thus considered non-responders. Interestingly, 22.22% of the IgG mice exhibited a decrease from their max tumor size after injection 6 (day 11), despite the lack of therapeutic intervention. Figure 1b also highlights the variation in starting tumor size, an inherent limitation due to the nature of the model.

To overcome this variation, each tumor was normalized to its respective baseline to assess the relative percent change in tumor size. Figure 1c mirrors the trend seen in Figure 1a, accounting for differences in initial tumor size. Initially, anti-PD1-treated tumors were slightly larger, starting at an average tumor size of 101.1 mm^2^ compared to IgG at 91.17 mm^2^. At day 7 (injection 4), both groups’ averages were nearly identical (125.81 mm^2^ and 129.37 mm^2^), but average percentage changes of 27% ± 17% for PD1 and 51% ± 8% for IgG from baseline were seen. By day 18 (injection 9), there was a large difference in average percentage change, with the anti-PD1 group exhibiting a 24% ± 21% increase from initial tumor size, and IgG-treated tumors showing an increase of 126% ± 37% from initial tumor size, though this difference did not reach statistical significance (*p* = 0.3671, Bonferroni-corrected).

Figure 1d,e depicts the morphological changes of cSCC between mice from the PD1 and IgG treatment groups. In Figure 1d, the anti-PD1-treated mouse’s tumor is initially above a benign papilloma but increased from 11.0 mm to 13.5 mm over the treatment course. Conversely, Figure 1e illustrates the IgG-treated tumor doubling in length from 7.1 mm to 14.4 mm.

### 3.2. Combinatory Anti-PD1 and Radiation Therapy Slows Growth Rate in Both Anti-PD1 and IgG-Treated Mice

**Figure 2:**
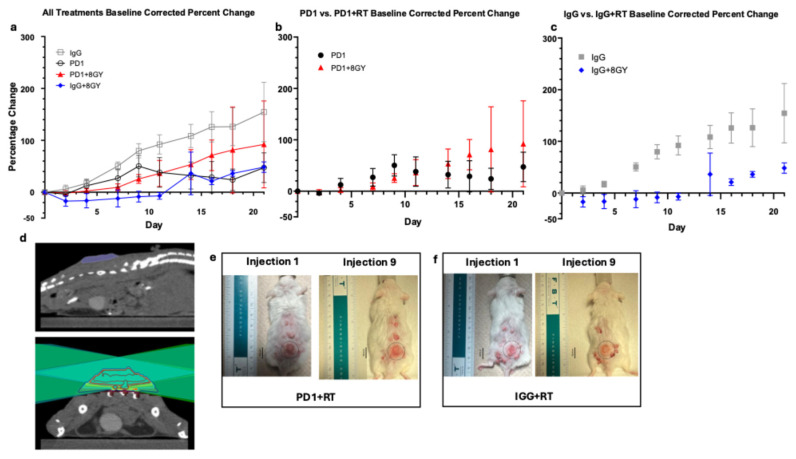
(**a**) Average baseline-corrected percent change of Anti-PD1 (*n* = 10), IgG (*n* = 8), Anti-PD1 + RT (*n* = 4), and IgG + RT (*n* = 3)-treated tumors. Error bars represent SEM. (**b**) Average baseline-corrected percent change of anti-PD1 and anti-PD1 + RT-treated tumors. Error bars represent SEM. (**c**) Average baseline-corrected percent change of IgG and IgG + RT-treated tumors. Error bars represent SEM. (**d**) Top image is tumor in sagittal view; bottom image is treatment plan shown in axial view. Topographical lines describe dose of radiation given to varying regions of tumor. Dark red lines represent regions that received 120% dose of 9.6 Gy, red represents 100% dose (8.0 Gy), yellow represents 80% dose (6.0 Gy), cyan represents 50% dose (4.0 Gy), and navy blue represents 20% dose (1.6 Gy) (**e**) Visual progression of cSCC lesion after radiation and 3 weeks of anti-PD1 treatment. Grey circle represents area of interest with standardize diameter of 17 mm. (**f**) Visual progression of cSCC lesion after radiation and 3 weeks of IgG treatment. Grey circle represents area of interest with standardize diameter of 17 mm.

To evaluate the additive effects of radiation therapy (RT), mice received 8 gray (Gy) of targeted RT in a single fraction prior to a nine-injection course of anti-PD1 or IgG. Figure 2a shows the percent change in tumor size across all treatment groups. IgG control mice exhibited the largest percent change of all groups (154% ± 57% on day 21), indicating uncontrolled tumor growth. In contrast, IgG + RT mice showed minimal or negative growth at days 9 to 11 (injections 5–6) (Figure 2c), demonstrating that radiation significantly reduced tumor progression compared to IgG controls (*p* = 0.0097 and *p* = 0.0121, Bonferroni-corrected). Radiated mice exhibited similar percent changes on day 21 as PD1 monotherapy mice (48% ± 10% and 47% ± 29%, respectively), suggesting that radiation aids in reducing the rate of tumor growth but does not provide a synergistic benefit, as combination therapy did not significantly enhance responses (*p* = 0.6877).

Despite having a larger starting size (mean of 145.14 mm^2^ for PD1 + RT and 101.10 mm^2^ for PD1), PD1 + RT mice show a slightly reduced rate of percent change in the first half of treatment as opposed to PD1 alone (Figure 2b). On day 9 (injection 5), percent growth was lower in PD1 + RT (25% ± 9%) than PD1 alone (50% ± 21%). However, by the end of treatment, there was no significant difference between percentage change of PD1 monotherapy and PD1 + RT combinatory treatment, with average percent changes of 47% ± 29% and 92% ± 84%, respectively (*p* = 0.6877).

Looking at the effects of radiation alone (Figure 2c), on day 21, there was a large difference between the percent change of IgG (154% ± 57%) and IgG + RT (48% ± 10%); however, this was not statistically significant. Comparing PD1 + RT and IgG + RT, we see that IgG + RT is a lower percent change than PD1 + RT (48% and 92% respectively), suggesting that PD1 and RT monotherapy might be similarly effective.

PD1 + RT had the lowest survival probability (50%), likely attributed to side effects of combinatory treatment, larger initial starting size, and possible spinal dose irradiation; thus, these mice were quicker to reach ethical endpoints (greater than 20 mm in a single dimension). While RT treatment plans were designed to minimize spinal dose, tumor location on the dorsal flank made it challenging to completely avoid spinal irradiation. Some mice received a large spinal dose, which may have contributed to earlier ethical endpoints. In addition to treatment with 8 Gy, mice were also treated with 12 Gy; however, this treatment regimen was terminated due to the intensity of radiation, resulting in a 0% survival rate in both IgG + 12 Gy and PD1 + 12 Gy mice. 

In Figure 2e,f we can visualize the very minimal increases in length over the duration of 3 weeks of immunotherapy treatment in both PD1 + RT and IgG + RT mice. In Figure 2e, the PD1 + RT mouse starts treatment at a length of 11.5 mm and a width of 10.5 mm and ends treatment at 10.9 mm by 10.9 mm. Figure 2f shows a similar trend with the IgG + RT tumor beginning with a length of 10.0 mm by 9.0 mm, ending at 10.9 mm by 10.5 mm in width.

### 3.3. Sarcomatous Morphology Associated with Irradiated Tumors and Metastasis Incidence in Sentinel Lymph Nodes

**Figure 3:**
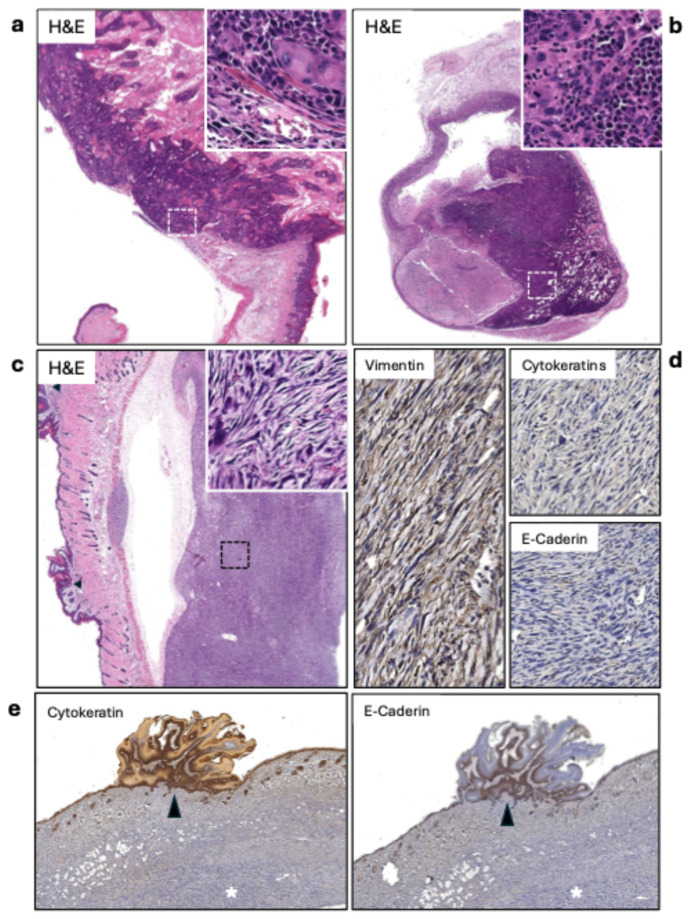
(**a**) Keratinizing squamous cell carcinoma (SCC) (20×). The inset (400×) shows the carcinoma infiltrating the muscle layer. (**b**) Lymph node metastasis of the SCC shown in A. The lesion covers about 80% of the lymph node’s area (20×). The inset (400×) displays the highly atypical squamous cells infiltrating the node. (**c**) Sarcomatous tumor. The lesion is separated from the skin due to retraction during fixation. The inset shows atypical spindle-shaped cells. On the right side of the image, papillomatous lesions can be observed (arrowheads). (**d**) The sarcomas tested positive for vimentin and negative for cytokeratins and e-cadherin. (**e**) Positive controls for cytokeratins and E-cadherin (20×). The papillomatous growths are positive for cytokeratins and E-cadherin (arrowheads), while the underlying sarcoma is negative for both.

After treatment, all tumor samples harvested were verified as cSCC via histochemistry using cytokeratin and visual morphology by pathologist analysis (Figure 3a). Among the tumors, irradiated tumors were found to express a different morphology than traditional cSCC (Figure 3c). They presented as fibroblastic with spindle-like cells with an absence of cytokeratin and e-cadherin expression and were positive for vimentin (Figure 3d). This sarcoma phenotype was observed only in irradiated mice, occurring in 33.3% of IgG + RT tumors and 50.0% of PD1 + RT tumors (Table 1). Mice also developed benign papillomas during the tumorigenesis process. Interestingly, in one case (Figure 3e), the papilloma was positive for cytokertatins and E-cadherin, whereas the sarcoma underneath was negative for both. We cannot conclude that this phenotype is derived from radiation or if it occurred before the radiation treatment, and the presence of this phenotype only in irradiated mice suggests a potential association of radiation toward the development of this phenotype in these spontaneous cSCC tumors, which would need to be confirmed in a larger sample size.

Sentinel draining lymph nodes were identified using the fluorescent agent tilmanocept, which was used and harvested for pathology. Figure 3b displays a representative case of metastasis, with atypical squamous cells infiltrating about 80% of the lymph node. Of the sentinel lymph nodes harvested, 31.82% were cases of metastasis (Table 2). Of these seven cases of metastasis, there was no significant correlation between the treatment group and the development of metastasis in lymph nodes, occurring in every treatment type, including mice before treatment. It is important to note that mice in this model can develop multiple primary cSCC lesions. While we targeted the largest tumor for radiation treatment and used tilmanocept lymphatic mapping to identify sentinel nodes draining from this primary site, we cannot definitively exclude the possibility that smaller secondary tumors also contributed to the metastatic burden observed in regional lymph nodes.

### 3.4. Anti-PD1 and Radiation Combinatory Treatment Significantly Increases Immune Infiltrate in Tumor Center

To evaluate potential treatment-associated differences in immune infiltrate, we conducted a preliminary analysis of tumors from individual mice representing each treatment group (*n* = 1 per treatment, *n* = 5 fields of view per tumor). Tumors were stained for the following immune cells: CD4+ Helper T Cells, CD8+ Cytotoxic T Cells, and CD68+ Macrophages. The expression of those cells was normalized by the cell count (cell detection via DAPI channel) in each region. *p*-values represent comparisons of average FOV measurements between individual mice using Welch’s *t*-test and are reported without being adjusted for multiple comparisons. Statistical comparisons based on FOVs from a single biological specimen per treatment group are used to make exploratory observations but are insufficient to support definitive conclusions.

#### 3.4.1. CD8

**Figure 4:**
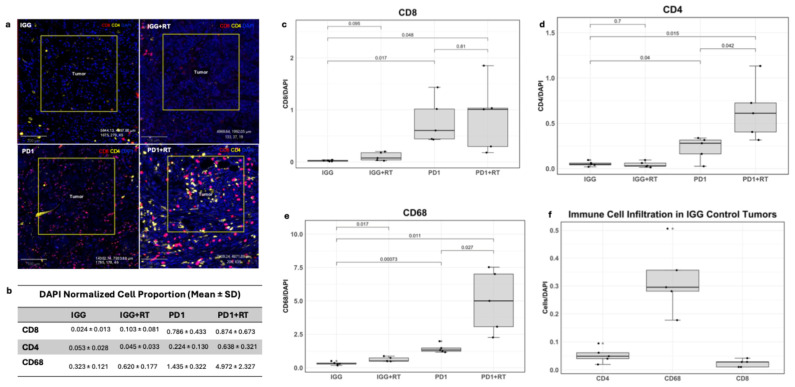
(**a**) Images of multiplex slides, stained red for CD8+ cells, stained yellow for CD4+, and DAPI stained in blue. (**b**) Average normalized proportion of immune infiltrating cells in the tumor center. (**c**) Box plot of normalized CD8+ cell proportion in the tumor center across treatments. (**d**) Box plot of CD4+ cell proportion in the tumor center across tumor treatments. (**e**) Box plot of CD68+ cell proportion in the tumor center across tumor treatments. (**f**) Normalized counts of each immune cell in the IgG-treated tumor.

In Figure 4a, both the anti-PD1 and anti-PD1 + RT-treated tumors showed elevated normalized CD8+ cell counts compared to the IgG control tumor in this preliminary single-specimen analysis. Quantification of immunofluorescence showed a 32.75% increase in the anti-PD1 monotherapy tumor and a 36.42% increase in the anti-PD1 + RT tumor compared to the IgG control (*p* = 0.017 and *p* = 0.048, respectively, Welch’s *t*-test). Radiation alone did not increase CD8+ cell infiltration (*p* = 0.095). In the tumor periphery, only the PD1 + RT group saw an increase in CD8+ cells compared to the IgG control (*p* = 0.016).

#### 3.4.2. CD4

Both anti-PD1 and anti-PD1 + RT-treated tumors also showed increases in CD4+ T helper cell infiltration compared to IgG (Figure 4a,b,d) (*p* = 0.04 and *p* = 0.015, respectively). CD4+ infiltration was 284.82% higher in the anti-PD1 + RT tumor than in anti-PD1 alone (*p* = 0.042). At the tumor periphery, only the IgG + RT tumor showed higher CD4+ levels compared to the control (*p* = 0.001)

#### 3.4.3. CD68

Overall, CD68+ cells were the most prevalent immune population at baseline and across all tumor treatments (Figure 4b,f). The anti-PD1 + RT tumor showed the largest increase in CD68+ cells (*p* = 0.011) but also the largest variability (SD = 2.329; Figure 4b,d). CD68+ infiltration was also higher in anti-PD1 + RT compared to anti-PD1 monotherapy (*p* = 0.027). In the immunotherapy alone group, only CD68+ cells were increased (*p* = 0.00073 compared to the IgG control). These increases were also seen in the tumor periphery, with all treatment tumors exhibiting elevated CD68+ levels compared to the IgG control (IgG vs. PD1: *p* = 0.0035; IgG vs. PD1 + RT: *p* = 0.00025, PD1 vs. PD1 + RT: *p* = 0.00021).

These preliminary findings suggest treatment-associated differences in immune infiltrate; however, biological replication with additional mice is needed to validate these observations.

## 4. Discussion

Prior to this study, there were no reports on treating spontaneous cSCC generation in a preclinical model with combined immunotherapy and radiation. Our findings demonstrate a trend suggesting that anti-PD1 therapy may slow the progression of cSCC tumor growth, though this did not reach statistical significance (*p* = 0.0775). In addition, it did not result in the complete regression of cSCC, with most mice showing continued tumor progression. A total of 40% of anti-PD1-treated mice exhibited reductions of 10% from peak tumor size; the overall reduction was minimal, and the variability in response resulted in a lack of statistical significance. In addition, radiation therapy prior to anti-PD1 treatment did not significantly enhance therapeutic response compared to anti-PD1 monotherapy.

cSCC is one of the most highly mutated cancers but is typically associated with low rates of metastasis (3–5%) and high survival rates [20]. Even though metastatic cSCC is relatively rare in humans, we saw a much more frequent occurrence, with around 30% of treated mice exhibiting metastasis in the sentinel lymph node. The high metastasis rate was independent of the treatment group, reinforcing the aggressive nature of this model and its utility in studying high-risk cSCC.

Even in the context of a limited anti-PD1 response, our preliminary analysis suggests that anti-PD1 therapy may enhance CD8+, CD4+, and CD68+ cell infiltration into tumors. Ideally, the radiation in supplement to the anti-PD1 therapy would act synergistically to enhance the immune infiltrate within the tumor microenvironment. Potential synergistic effects were observed in both CD4+ T helper cells and CD68+ macrophages, but not in the CD8+ T cells, with the combined radioimmunotherapy yielding larger increases than the monotherapy comparisons. However, additional biological replicates are needed to confirm these exploratory observations. In other cancer mouse models, radioimmunotherapy has also been shown to increase CD8+ T cell infiltration in a mouse model of eSCC [21]. In humans, studies report that high intra-tumoral CD8+ infiltration in immunotherapy patients is significantly associated with better overall survival [22]. Studies have also found tumor-associated macrophages (TAMs), differentiated macrophages that act in an immune-suppressive manner within the microenvironment and are associated with worse outcomes in multiple cancers [23,24,25]. The prevalence of CD68+ macrophages observed within the tumor center and periphery may explain the modest therapeutic response seen in our study; however, further histological markers should be used to distinguish between anti-tumoral and immunosuppressive phenotypes.

Clinical trials using cemiplimab as neoadjuvant therapy on resectable stage II to IV cSCC saw a complete response in 51% of patients [16]. About 25% of patients in this trial did not show a complete or major response, with more than 10% residual viable tumor cells in the surgical specimen [16]. Of these patients, 62% had a partial response, 20% had stable disease, and 10% had progressive disease [16]. Similarly, in a recurrent/metastatic disease trial, a 47.2% response rate was demonstrated [15]. An extended follow-up of Australian patients treated on the EMPOWER-CSCC-1 trial confirmed the durability of these responses, with a 5-year progression-free survival of 48% and a 5-year overall survival of 60%, but also demonstrated persistent non-response in the remaining ~40–50% of patients [26].

Overall, this model does not mirror the robust responses seen in most human patients, who generally respond to immunotherapy, but could represent the subpopulation of high-risk cSCC with delayed or modest responses [26,27]. The mechanisms underlying the limited response observed in this model remain to be fully characterized. If such parallels are established, this model could serve as a platform for investigating resistance mechanisms and testing combination therapeutic strategies.

Future investigations can explore the response rate to anti-PD1 inhibition in other models of cSCC. The generation of cSCC using the DMBA/TPA model in alternative genetic backgrounds such as C57/Black6 may demonstrate differential immunologic responses to treatment. In our model, we utilized FVB/NJ mice, which were bred to be sensitive to both histamine diphosphate and the B strain of the Friend leukemia virus [28]. Overall, the different background strains of mice yield different MHC haplotypes, FVB/NJ being categorized as MHC haplotype H2-kq and C57BL/6 as H2-kb. C57BL/6 mice have shown good responses to immunotherapy in preclinical models of melanoma, lung, and head and neck cancer [29,30,31]. Employing this background strain of mice could improve the therapeutic response in our model. In addition, our FVB/NJ model exhibited a high frequency of severe inflammation, blocking of lymphatic channels, disease dissemination to lymph nodes, and incidence of lymphoma. Additional alternative cell line-derived preclinical models could be implemented, such as the orthotopic injection of SCC7 into the dermis or implantation of cSCC cell line-derived xenografts in humanized mice [32,33]. However, their implementation in testing the effectiveness of anti-PD1 therapy remains limited because they require implantation into immunodeficient hosts [32,34].

Within this experiment, there were various challenges that contributed to the large variation and lack of statistical significance seen in the data. The use of carcinogenic chemicals to spontaneously generate cSCC has various positive and negative attributes. The spontaneous generation of tumors resulted in a more heterogenous background of lesions, which is more clinically representative. However, this heterogeneity in development, coupled with the large variation, morphology, and aggressiveness we saw in the growth rate of the tumors, results in difficulty standardizing the experiment.

Particularly, it is challenging to standardize cSCC tumor size prior to treatment as the tumors develop at varying rates—anywhere between 2 and 6 months of TPA application. As a result, mice are treated once tumors progress to a reasonable threshold, usually in batches of two to three mice at a time. This limitation can lead to batch effects and introduce potential confounding variables that can influence the data. Tumors also vary in their morphology, with the primary stages of cSCC being difficult to distinguish between benign papillomas and/or the scabs they leave behind, specifically when lesions are less than 5 mm. In addition, once the cSCC lesions generate, they grow at varying speeds. We observed that tumors that generated two months after TPA application were usually faster in tumor progression as opposed to tumors that were generated after 6 months of TPA application. It is also necessary to standardize the methods of identifying carcinogenic lesions visually, as biopsies are not reasonable in this model. Biopsies may potentially induce an immune response or ulceration, and there exists a short time frame in which treatment needs to be initiated. Given this, experience identifying the cSCC lesions is necessary, along with pathological conformation after treatment completion.

Initial group sizes varied and mortality/ethical endpoints further reduced sample sizes at later timepoints, complicating longitudinal analysis and contributing to the high variability observed in the data. This limited statistical comparisons and the ability to detect treatment differences statistically. Larger cohorts are needed to definitively establish treatment effects and response patterns.

Inherent differences between mice and human immune systems may limit the direct clinical translation of findings. While spontaneous tumor generation preserves the clinically relevant interactions of the tumor microenvironment, they are not fully translatable to human patients. Differences in MHC molecules, cytokine networks, and immune cell infiltration between mice and humans may influence both responses and resistance to treatment.

We also observed a frequent occurrence of metastasis and disease in many lymph nodes. One mouse was diagnosed with lymphoma, and many others exhibited inflammation, metastasis, and or necrosis within their lymph nodes, which further contributed to the progression of disease.

## 5. Conclusions

Previously, a spontaneously generated cSCC preclinical model had not been treated with immunotherapy and radiation to determine treatment efficacy. Our findings demonstrate that while anti-PD1 therapy slows the progression of cSCC tumor growth, it does not result in the complete regression of cSCC, with most mice showing continued tumor progression. While 40% of anti-PD1-treated mice exhibited reductions from peak tumor size, the overall reduction was minimal and the variability in response resulted in the lack of statistical significance. Radiation monotherapy was similarly effective as anti-PD1 in stabilizing tumor size. The nature of the spontaneous tumor generation model yields variability that mirrors the clinical heterogeneity observed in human patients, highlighting its clinical relevance. In addition, the limited response to anti-PD1 treatment demonstrates the potential for utilizing this preclinical model to study the mechanisms of limited immunotherapy response in cSCC, a population that continues to require more treatment options.

## Figures and Tables

**Table 1:** Percent incidence of sarcoma morphology in each treatment group. Mice that died before tumor harvest were excluded from pathological analysis.

	Number (%) *n* = 20
Total Sarcoma Morphology	3(15.0)
IgG (*n* = 5)	0 (0.0)
Anti-PD1 (*n* = 7)	0 (0.0)
IgG + RT (*n* = 3)	1 (33.3)
Anti-PD1 + RT (*n* = 5)	2 (40.0)

**Table 2:** Percent of metastasis in lymph nodes among all tumors and incidences of metastasis across treatment groups.

	Number (%) *n* = 22
Metastasis	7 (31.82)
IgG	1 (14.29)
Anti-PD1	2 (28.57)
IgG + RT	1 (14.29
Anti-PD1 + RT	2 (28.27)
No Treatment	1 (14.29)
No Metastasis	15 (68.18)

## Data Availability

Data is available upon request.

## Abbreviations

The following abbreviations are used in this manuscript:

cSCC	Cutaneous squamous cell carcinoma
RT	Radiation Therapy
PD1	Programmed cell death protein 1
IgG	Immunoglobulin G
Gy	Gray
DMBA	7,12-dimethylbenzo[a]anthracene
TPA	12-O-tetradecanoylphorbol-13-acetate

## References

[1] Hosseini T.M., Park S.J., Guo T. (2024). The Mutational and Microenvironmental Landscape of Cutaneous Squamous Cell Carcinoma: A Review. Cancers.

[2] Waldman A., Schmults C. (2019). Cutaneous Squamous Cell Carcinoma. Hematol. Clin. N. Am..

[3] Wang M., Gao X., Zhang L. (2024). Recent global patterns in skin cancer incidence, mortality, and prevalence. Chin. Med J..

[4] Corchado-Cobos R., García-Sancha N., González-Sarmiento R., Pérez-Losada J., Cañueto J. (2020). Cutaneous Squamous Cell Carcinoma: From Biology to Therapy. Int. J. Mol. Sci..

[5] Fania L., Didona D., Di Pietro F.R., Verkhovskaia S., Morese R., Paolino G., Donati M., Ricci F., Coco V., Ricci F. (2021). Cutaneous Squamous Cell Carcinoma: From Pathophysiology to Novel Therapeutic Approaches. Biomedicines.

[6] Que S.K.T., Zwald F.O., Schmults C.D. (2018). Cutaneous squamous cell carcinoma: Incidence, Risk Factors, Diagnosis, and Staging. J. Am. Acad. Dermatol..

[7] Jung K., Narwal M., Min S.Y., Keam B., Kang H. (2020). Squamous cell carcinoma of head and neck: What internists should know. Korean J. Intern. Med..

[8] Burton K.A., Ashack K.A., Khachemoune A. (2016). Cutaneous Squamous Cell Carcinoma: A Review of High-Risk and Metastatic Disease. Am. J. Clin. Dermatol..

[9] Grob J.-J., Gonzalez R., Basset-Seguin N., Vornicova O., Schachter J., Joshi A., Meyer N., Grange F., Piulats J.M., Bauman J.R. (2020). Pembrolizumab Monotherapy for Recurrent or Metastatic Cutaneous Squamous Cell Carcinoma: A Single-Arm Phase II Trial (KEYNOTE-629). J. Clin. Oncol..

[10] Dantoing E., Piton N., Salaün M., Thiberville L., Guisier F. (2021). Anti-PD1/PD-L1 Immunotherapy for Non-Small Cell Lung Cancer with Actionable Oncogenic Driver Mutations. Int. J. Mol. Sci..

[11] Shalhout S.Z., Emerick K.S., Kaufman H.L., Miller D.M. (2021). Immunotherapy for Non-melanoma Skin Cancer. Curr. Oncol. Rep..

[12] Lei Q., Wang D., Sun K., Wang L., Zhang Y. (2020). Resistance Mechanisms of Anti-PD1/PDL1 Therapy in Solid Tumors. Front. Cell Dev. Biol..

[13] Seidel J.A., Otsuka A., Kabashima K. (2018). Anti-PD-1 and Anti-CTLA-4 Therapies in Cancer: Mechanisms of Action, Efficacy, and Limitations. Front. Oncol..

[14] Anwaar S., Ashraf A., Jahfali S., Yunis J., Cruz J.L.G., Wells J.W. (2025). Immunomodulation of UVB-induced regulatory T cells prevents the establishment of squamous cell carcinoma. J. Immunother. Cancer.

[15] Hughes B.G., Guminski A., Bowyer S., Migden M.R., Schmults C.D., Khushalani N.I., Chang A.L.S., Grob J.-J., Lewis K.D., Ansstas G. (2024). A phase 2 open-label study of cemiplimab in patients with advanced cutaneous squamous cell carcinoma (EMPOWER-CSCC-1): Final long-term analysis of groups 1, 2, and 3, and primary analysis of fixed-dose treatment group 6. J. Am. Acad. Dermatol..

[16] Gross N.D., Miller D.M., Khushalani N.I., Divi V., Ruiz E.S., Lipson E.J., Meier F., Su Y.B., Swiecicki P.L., Atlas J. (2022). Neoadjuvant Cemiplimab for Stage II to IV Cutaneous Squamous-Cell Carcinoma. N. Engl. J. Med..

[17] Rischin D., Porceddu S., Day F., Brungs D.P., Christie H., Jackson J.E., Stein B.N., Su Y.B., Ladwa R., Adams G. (2025). Adjuvant Cemiplimab or Placebo in High-Risk Cutaneous Squamous-Cell Carcinoma. N. Engl. J. Med..

[18] Aoto Y., Okumura K., Hachiya T., Hase S., Wakabayashi Y., Ishikawa F., Sakakibara Y. (2018). Time-Series Analysis of Tumorigenesis in a Murine Skin Carcinogenesis Model. Sci. Rep..

[19] Abel E.L., Angel J.M., Kiguchi K., DiGiovanni J. (2009). Multi-stage chemical carcinogenesis in mouse skin: Fundamentals and applications. Nat. Protoc..

[20] Dessinioti C., Pitoulias M., Stratigos A.J. (2021). Epidemiology of advanced cutaneous squamous cell carcinoma. J. Eur. Acad. Dermatol. Venereol..

[21] Yin Z., Zhang H., Zhang K., Yue J., Tang R., Wang Y., Deng Q., Yu Q. (2025). Impacts of combining PD-L1 inhibitor and radiotherapy on the tumour immune microenvironment in a mouse model of esophageal squamous cell carcinoma. BMC Cancer.

[22] Li F., Li C., Cai X., Xie Z., Zhou L., Cheng B., Zhong R., Xiong S., Li J., Chen Z. (2021). The association between CD8+ tumor-infiltrating lymphocytes and the clinical outcome of cancer immunotherapy: A systematic review and meta-analysis. eClinicalMedicine.

[23] Seminerio I., Kindt N., Descamps G., Bellier J., Lechien J.R., Mat Q., Pottier C., Journé F., Saussez S. (2018). High infiltration of CD68+ macrophages is associated with poor prognoses of head and neck squamous cell carcinoma patients and is influenced by human papillomavirus. Oncotarget.

[24] Zhang Q.W., Liu L., Gong C.-Y., Shi H.-S., Zeng Y.-H., Wang X.-Z., Zhao Y.-W., Wei Y.-Q. (2012). Prognostic Significance of Tumor-Associated Macrophages in Solid Tumor: A Meta-Analysis of the Literature. PLoS ONE.

[25] Dong Q., Zhang Z., Li S., Liang L. (2025). Mechanisms of immunotherapy in cutaneous squamous cell carcinoma in the tumor microenvironment. Front. Immunol..

[26] Bennett C., McLean L.S., Lim A.M., Bressel M., Guminski A., Hughes B.G., Bowyer S.E., Stein B., Rischin D. (2025). Extended follow-up outcomes of patients with advanced cutaneous squamous cell carcinoma treated with cemiplimab on EMPOWER-CSCC-1: A retrospective cohort study. J. Am. Acad. Dermatol..

[27] García-Sancha N., Corchado-Cobos R., Bellido-Hernández L., Román-Curto C., Cardeñoso-Álvarez E., Pérez-Losada J., Orfao A., Cañueto J. (2021). Overcoming Resistance to Immunotherapy in Advanced Cutaneous Squamous Cell Carcinoma. Cancers.

[28] Wong K., Bumpstead S., Van Der Weyden L., Reinholdt L.G., Wilming L.G., Adams D.J., Keane T.M. (2012). Sequencing and characterization of the FVB/NJ mouse genome. Genome Biol..

[29] Phadke M.S., Chen Z., Li J., Mohamed E., Davies M.A., Smalley I., Duckett D.R., Palve V., Czerniecki B.J., Forsyth P.A. (2021). Targeted Therapy Given after Anti–PD-1 Leads to Prolonged Responses in Mouse Melanoma Models through Sustained Antitumor Immunity. Cancer Immunol. Res..

[30] Li H.Y., McSharry M., Bullock B., Nguyen T.T., Kwak J., Poczobutt J.M., Sippel T.R., Heasley L.E., Weiser-Evans M.C., Clambey E.T. (2017). The Tumor Microenvironment Regulates Sensitivity of Murine Lung Tumors to PD-1/PD-L1 Antibody Blockade. Cancer Immunol. Res..

[31] Jang J.Y., Lee B., Huang M., Seo C., Choi J., Shin Y.S., Woo H.G., Kim C. (2024). Immune checkpoint inhibitor monotherapy is sufficient to promote microenvironmental normalization via the type I interferon pathway in *PD-L1*-expressing head and neck cancer. Mol. Oncol..

[32] Hsu C.-Y., Yanagi T., Maeda T., Nishihara H., Miyamoto K., Kitamura S., Tokuchi K., Ujiie H. (2023). Eribulin inhibits growth of cutaneous squamous cell carcinoma cell lines and a novel patient-derived xenograft. Sci. Rep..

[33] Li C., Sun C., Lohcharoenkal W., Ali M.M., Xing P., Zheng W., Görgens A., Gustafsson M.O., EL Andaloussi S., Sonkoly E. (2023). Cutaneous squamous cell carcinoma-derived extracellular vesicles exert an oncogenic role by activating cancer-associated fibroblasts. Cell Death Discov..

[34] Capasso A., Lang J., Pitts T.M., Jordan K.R., Lieu C.H., Davis S.L., Diamond J.R., Kopetz S., Barbee J., Peterson J. (2019). Characterization of immune responses to anti-PD-1 mono and combination immunotherapy in hematopoietic humanized mice implanted with tumor xenografts. J. Immunother. Cancer.

